# Insufficient Generation of Mycobactericidal Mediators and Inadequate Level of Phagosomal Maturation Are Related with Susceptibility to Virulent *Mycobacterium tuberculosis* Infection in Mouse Macrophages

**DOI:** 10.3389/fmicb.2016.00541

**Published:** 2016-04-18

**Authors:** Hyo-Ji Lee, Hyun-Jeong Ko, Yu-Jin Jung

**Affiliations:** ^1^Department of Biological Sciences and BIT Medical Convergence Graduate Program, Kangwon National UniversityChuncheon, South Korea; ^2^College of Pharmacy, Kangwon National UniversityChuncheon, South Korea

**Keywords:** macrophage, *Mycobacterium tuberculosis*, nitric oxide (NO), phagosomal maturation, reactive oxygen species (ROS), susceptibility

## Abstract

Tuberculosis is caused by *Mycobacterium tuberculosis* infection, and it remains major life-threatening infectious diseases worldwide. Although, *M. tuberculosis* has infected one-third of the present human population, only 5–10% of immunocompetent individuals are genetically susceptible to tuberculosis. All inbred strains of mice are susceptible to tuberculosis; however, some mouse strains are much more susceptible than others. In a previous report, we showed that Th1-mediated immunity was not responsible for the differential susceptibility between mouse models. To examine whether these susceptibility differences between inbred mouse strains are due to the insufficient production of effector molecules in the early stage of innate immunity, we investigated mycobacteriostatic function of bone marrow-derived macrophages (BMDMs) in resistant (BALB/c and C57BL/6) and susceptible strains (DBA/2) that were infected with virulent *M. tuberculosis* (H37Rv) or attenuated *M. tuberculosis* (H37Ra). The growth rate of virulent *M. tuberculosis* in infected cells was significantly higher in DBA/2 BMDMs, whereas the growth of the attenuated strain was similar in the three inbred mouse BMDM strains. In addition, the death rate of *M. tuberculosis*-infected cells increased with the infectious dose when DBA/2 BMDMs were infected with H37Rv. The intracellular reactive oxygen species level was lower in DBA/2 BMDMs that were infected with virulent *M. tuberculosis* at an early post-infection time point. The expression levels of phagosomal maturation markers, including early endosomal antigen-1 (EEA1) and lysosome-associated membrane protein-1 (LAMP-1), were significantly decreased in DBA/2 BMDM that were infected with virulent *M. tuberculosis*, whereas IFNγ-treatment restored the phagosomal maturation activity. The nitric oxide (NO) production levels were also significantly lower in DBA/2 BMDMs that were infected with virulent H37Rv at late post-infection points; however, this was not observed with the attenuated H37Ra strain. Furthermore, IFNγ-treatment rescued the low NO production level and insufficient *M. tuberculosis* growth control of DBA/2 BMDMs to the same level as of both resistant strains. The secreted TNF-α and IL-10 level were not significantly different between strains. Therefore, our findings suggest that DBA/2 BMDMs may have defects in the phagosomal maturation process and in inflammatory mediator production, as they showed innate immune defects when infected with the virulent, but not attenuated *M. tuberculosis* strain.

## Introduction

Tuberculosis (TB) is an infectious bacterial disease that has infected over one-third of the world’s population. However, only 5–10% of the population is genetically susceptible to tuberculosis (Eurosurveillance editorial team, 2013). After inhaling droplets containing tubercle bacilli, the disease occurs when the infection develops into a progressive pathology in the lungs (O’Garra et al., 2013). *Mycobacterium tuberculosis* is a facultative intracellular pathogen that is the causative agent of TB. After *M. tuberculosis* recognized, macrophages serve as the first line of defense against bacterial pathogens. These cells recognize and kill invading pathogens and secrete inflammatory cytokines or reactive nitrogen intermediates (RNIs) to activate several downstream signaling pathways that affect the ability of mycobacteria to survive intracellularly (Chan et al., 1992). However, *M. tuberculosis* has developed various strategies, including phagosome–lysosome fusion interference, phagosome acidification inhibition, inflammatory immune suppression, and host cell death modulation to survive and replicate inside macrophages during phagocytosis (Poirier and Av-Gay, 2012). Several *M. tuberculosis* products play roles in the inhibition of phagosomal maturation, including LAM, phosphatase, and ESAT-6 (Fratti et al., 2003; Xu et al., 2007; Puri et al., 2013). Recent studies have shown that in virulent *M. tuberculosis*, the glycosylated phosphatidylinositol ManLAM interferes with the phagosomal acquisition of lysosomal cargo and syntaxin 6, which participates in vesicular trafficking between the *trans*-Golgi network and the endocytic pathway (Fratti et al., 2003). Furthermore, IL-10, an anti-inflammatory cytokine, blocks phagosomal maturation in *M. tuberculosis*-infected human macrophages through p38 MAPK-independent mechanism (O’Leary et al., 2011). However, the process of phagosomal maturation arrest that is mediated by *M. tuberculosis* is not fully understood.

All inbred mouse strains exhibit susceptibility to infection with *M. tuberculosis* and develop progressive disease in the lungs (North and Jung, 2004; Gupta and Katoch, 2009). However, inbred mouse strains differ substantially in their degree of susceptibility to *M. tuberculosis* infection (Medina and North, 1998). Recent studies have characterized mouse strains as either susceptible (DBA/2, C3H, CBA, and 129Sv) or resistant (C57BL/6 and BALB/c) to intravenous (iv) inoculation with *M. tuberculosis*. Susceptibility was defined as early death, which was associated with progressive pathology in the lungs that resulted from the failure to control bacterial growth and a consequentially severe reduction in available surface area in the lungs for oxygen exchange (Medina and North, 1998). In addition, it has been demonstrated that C57BL/6 mice generate a much higher Th1 response than DBA/2 or BALB/c mice, suggesting that susceptibility is not determined by changes in Th1 cell-mediated immunity (Jung et al., 2009). The genes that are critical to determining susceptibility to *M. tuberculosis* infection have not been fully identified in either humans or mice. Although, tuberculosis resistance loci have been characterized in two mouse strains, DBA/2 and C57BL/6 (Marquis et al., 2009), research aimed to investigating the major genetic susceptibility factors in mouse models is ongoing. In this report, to verify the hypothesis that innate differences in the function of macrophages amongst inbred mouse strains could influence susceptibility to *M. tuberculosis* infection, we examined bone marrow-derived macrophages (BMDMs) in three strains that were infected with either low or very low levels of *M. tuberculosis*. Our findings revealed that virulent *M. tuberculosis* growth rates in infected cells were significantly higher in the DBA/2 BMDMs, while the growth rates of the attenuated strain were similar in the three inbred mouse BMDM strains. In addition, *M. tuberculosis*-induced cell death increased as the infection dose increased when DBA/2 BMDMs were infected with H37Rv, but not when they were infected with H37Ra. Reactive oxygen species (ROS) and nitric oxide (NO) were produced in response to the virulent *M. tuberculosis* strain, H37Rv, and the attenuated *M. tuberculosis* strain, H37Ra. Furthermore, treatment of IFN-γ returned the low NO production level and the insufficient *M. tuberculosis* growth control in the DBA/2 BMDMs to the same level that was observed in both resistant strains. Co-localization between H37Rv and lysosome-associated membrane protein-1 (LAMP-1) was significantly decreased in the DBA/2 BMDMs, and procathepsin D processing into its active enzyme form (cleaved cathepsin D) was significantly diminished in H37Rv-infected DBA/2 BMDMs. However, the levels of secreted TNF-α and IL-10 were not associated with differential susceptibility to infection with virulent *M. tuberculosis* in each BMDM. These findings demonstrate that DBA/2 macrophages are more susceptible than cells in other mouse strains because they display defects in the ability to generate innate immune mediators and to facilitate phagosomal maturation following infection with virulent *M. tuberculosis* strains, but not in response to infection with attenuated *M. tuberculosis* strains.

## Materials and Methods

### Mice

Five weeks-old male BALB/c, C57BL/6, and DBA/2 mice were purchased from Narabio (Nara Bio., Korea) for use in this study. All mice were housed under specific pathogen-free conditions with food and water provided *ad libitum*. All mice were sacrificed when they were 8–10 weeks-old to isolate bone marrow-derived cells. The animal use protocol was performed in accordance with the Institutional Animal Care and Use Committee of Kangwon National University (KIACUC).

### Bacterial Strains and Culture Conditions

The H37Rv and H37Ra *M. tuberculosis* strains were used as the virulent and attenuated strain types, respectively. The *M. tuberculosis* H37Rv and H37Ra strains were grown at 37°C in Middlebrook 7H9 broth (Difco Laboratories, Detroit, MI, USA) supplemented with 10% ADC (5% bovine albumin, 2% dextrose, 0.03% catalase, and 0.85% sodium chloride) and containing 0.2% glycerol. After 3 weeks of culture, the *M. tuberculosis* was harvested, adjusted to 1 × 10^7^ bacteria per 200 μl of stock solution, aliquoted, and maintained at -70°C until use. The *M. tuberculosis* H37Rv and H37Ra strains were then cultivated on Middlebrook 7H10 agar (Difco Laboratories, Detroit, MI, USA) supplemented with 10% OADC (0.06% oleic acid, 5% bovine albumin, 2% dextrose, 0.03% catalase, and 0.85% sodium chloride) and containing 0.5% glycerol. The viability of the *M. tuberculosis* cultures was measured as colony-forming units (CFUs) after cultures were plated at bacterial dilutions onto Middlebrook 7H10 agar plates.

### Isolation and Culture of Murine BMDMs

Femurs and tibias were dissected from mice, and the muscle was removed. Both ends of the bones were cut, and the bones were then flushed with RPMI1640 (Lonza, USA) using an 18-gage needle. The cells were centrifuged for 5 min at 1,000 rpm, and then resuspended in RPMI1640 containing 10% FBS (Lonza, USA) for 2 h. Non-adherent cells were collected and, centrifuged, and the red blood cells were removed. Cells were grown in culture dishes in the presence of 30% of the supernatant obtained from L929 cells as a source of M-CSF for 4 days. Non-adherent cells were removed using PBS washes, and the medium was replenished. Adherent cells were used the following day for infection experiments.

### *M. tuberculosis* Infections

Bone marrow-derived macrophages were infected with the *M. tuberculosis* H37Rv or H37Ra strain at an infection ratio of 0.1:1, 1:1, or 5:1. After 4 h of incubation at 37°C in 5% CO_2_ to facilitate bacterial uptake, the infected BMDMs were washed with PBS to remove any extracellular bacteria. To analyze the anti-bacterial activity of the BMDMs, the cells were stimulated with mouse recombinant IFN-γ (20 ng/ml, R&D Systems, USA) for 2 h either before or after infection for the indicated time points. For the kinetic growth assays, BMDMs were lysed using 0.1% saponin, and serial dilutions were seeded in Middlebrook 7H10 agar supplemented with OADC. The plates were then incubated at 37°C for 21 days.

### CFU Determination

A total of 2 × 10^5^ BMDMs/well were infected with the *M. tuberculosis* H37Rv or H37Ra strain for 4 h. The cells were then, washed and lysed using 0.1% saponin at the indicated points. *M. tuberculosis* was released from the cells by 0.1% saponin that was serially diluted in Middlebook 7H9 broth supplemented with ADC. A 50 μl volume obtained from three dilutions was plated on Middlebrook 7H10 agar with OADC triplicate. CFUs were counted after 21 days of incubation at 37°C.

### NO Assay

Nitric oxide was measured in BMDM culture supernatants by analyzing its reduction to NO_3_^-^ using an NO detection kit according to the manufacturer’s instructions (Intron-bio, Korea). This protocol is based on a diazotization (Griess method) assay. In brief, supernatants were collected from *M. tuberculosis*-infected BMDMs at the indicated time points, and then 100 μl of each sample was mixed with 50 μl of N1 buffer (with sulfanilamide in the reaction buffer) for 10 min at room temperature. After 10 min, the mixture was added to 50 μl of N2 buffer (with naphthylethylenediamine in the stabilizer buffer) for 10 min at room temperature, and the absorbance value was then measured between 520 and 560 nm using a plate reader. NO production was calculated using the standard curve of a nitrite standard solution.

### Enzyme Linked Immunosorbent Assay (ELISA)

Enzyme linked immunosorbent assay (ELISA) were performed on samples obtained from infected BMDM suspensions at the indicated time points according to the manufacturer’s protocols. Briefly, to detect murine TNF-α and IL-10 (Peprotech, Rocky Hill, CT, USA), the ELISA plates were coated with 1 μg/ml of a capture antibody overnight at room temperature. One hundred microliters of cell supernatant was used for each reaction, and either TNF-α or IL-10 was detected using secondary biotinylated anti-TNF-α or anti-IL-10 detection antibodies, respectively, followed by avidine peroxidase and an ABTS liquid substrate (Sigma-Aldrich, St. Louis, MO, USA). The plate was then read at an absorbance of 405 nm. Recombinant murine TNF-α and IL-10 were used as the standards.

### Cell Viability

Cell viability was determined after *M. tuberculosis* infection using trypan blue exclusion assays. BMDMs were seeded at 1 × 10^4^ cells/ml on 6-well plates and incubated at 37°C with 5% CO_2_. BMDMs were infected with H37Rv or H37Ra at a multiplicity of infection (MOI) of 0.1 or 1 for 4 h, and the cells were then washed with PBS. The numbers of viable cells were counted using a light microscope at various times after *M. tuberculosis* infection.

### Annexin V and Propidium Iodide (PI) Staining for Flow Cytometry

Bone marrow-derived macrophages (1 × 10^5^ cells) were infected with H37Rv *M. tuberculosis* at an MOI of 1 for 4 h, washed with PBS, and cultured for 12 or 72 h in 6-well plates at 37°C with 5% CO_2_. BMDMs were scraped, spun down, and washed with 1 ml of cold PBS. BMDMs were resuspended in Annexin V binding buffer containing 5 μl of Annexin V-FITC (BD Pharmingen^TM^, San Diego, CA, USA) and 5 μl of PI (Sigma-Aldrich, St. Louis, MO, USA) for 15 min at room temperature in the dark. After the cells were stained, they were washed once with binding buffer containing 4% paraformaldehyde and then analyzed on a BD-FACSCalibur instrument (BD-Biosciences, San Jose, CA, USA). Flow cytometric analysis was performed using CellQuest (BD-Biosciences) software.

### Lactate Dehydrogenase (LDH) Assay

Bone marrow-derived macrophages were incubated in 96-well plate and infected with H37Rv or H37Ra at MOI of 1 for 4 h. After then, cells were washed with PBS and incubated in RPMI1640 (containing 10% FBS) for 48 h. Lactate dehydrogenease (LDH) activity in the media was determined using LDH assay kit (Promega, Madison, WI, USA) according to the manufacturer’s protocols.

### Intracellular ROS Measurement

Intracellular ROS levels were measured using a fluorescence-based dye, 2′,7′-dichlorofluoresecin diacetate (DCFH-DA). In short, BMDMs were allowed to adhere to coverslips in 12-well plates before they were infected with *M. tuberculosis*. The cells were treated with RPMI 1640 medium containing 10 μM DCFH-DA for 20 min at 37°C with 5% CO_2_, washed twice with PBS and fixed in PBS containing 4% paraformaldehyde. Coverslips were mounted in Fluoromount-G^TM^ (SouthernBiotech, Birmingham, AB, USA) and examined using confocal microscopy [Olympus (FV1000 SPD)]. For flow cytometry, cells were rinsed with PBS and the fluorescence intensity of the DCF was measured using a FACSCalibur (BD Biosciences). The data were plotted and analyzed using CellQuest software.

### Immunofluorescence

Bone marrow-derived macrophages were allowed to adhere to coverslips in 12-well plates and the cells were then infected with fluorescein (FITC)-labeled *M. tuberculosis* H37Rv or H37Ra at an MOI of 5 for 4 h. After 4 h, the unbound *M. tuberculosis* was washed away, and the cells were cultured in complete RPMI 1640 without antibiotics until the indicated time points. Then, the cells were washed with PBS and fixed in PBS containing 4% paraformaldehyde overnight at 4°C. After the cells were fixed, they were permeabilized in 1% Triton X-100 for 10 min and incubated with Alexa Fluor^®^ 647 anti-mouse CD107a LAMP-1 (BioLegend, San Diego, CA, USA) or anti-EEA1-Alexa Fluor^®^ 647 (MBL, Woburn, MA, USA) for 2 h at room temperature. Coverslips were mounted in Fluoromount-G^TM^ (SouthernBiotech, Birmingham, AB, USA) and examined using confocal microscopy [Olympus (FV1000 SPD)].

### Western Blot Assay

Macrophages were infected with *M. tuberculosis* until the indicated time points. Western blot assays were performed as previously described (Lee et al., 2015). For the Western blot assays, anti-iNOS, anti-cathepsin D, and anti-actin antibodies were purchased from Santa Cruz Biotechnology (Dallas, TX, USA). The housekeeping protein, actin, was used to confirm that the well were equal loaded.

### Statistical Analysis

The data were obtained from independent experiments. The significance levels used for comparisons between samples were determined using ANOVA tests in GraphPad Prism 5 software (GraphPad Software, San Diego, CA, USA). Statistical significance is indicated as ^∗^*P* < 0.05; ^∗∗^*P* < 0.01; ^∗∗∗^*P* < 0.001 and n.s., not significant (*P* < 0.05).

## Results

### Susceptible Mouse BMDMs Allow Higher Bacterial Growth and Host Cell Death

**FIGURE 1:**
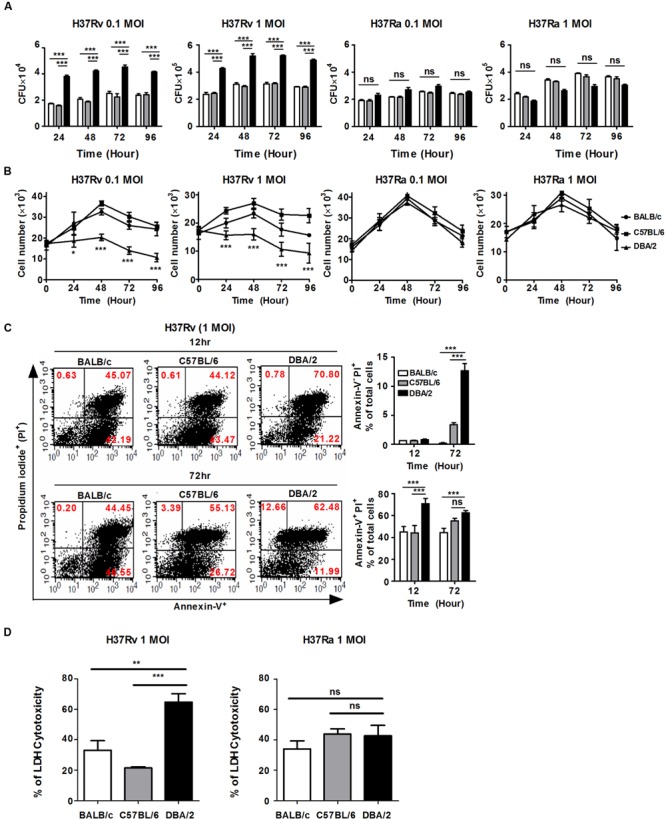
**H37Rv-infected DBA/2 BMDMs fail to control *M. tuberculosis* growth and host cell death. (A)** BMDMs obtained from each of the three mouse strains were seeded at 2 × 10^5^ cells/well and infected with virulent *M. tuberculosis* H37Rv or attenuated *M. tuberculosis* H37Ra (at MOIs of 0.1 and 1) for 4 h. The viability of intracellular bacteria was assayed based on the number of CFUs at indicated time points. The data are presented as the mean ± standard deviation of experiments that were perforemd in triplecate. **(B)** BMDM were seeded at 1 × 10^4^ cell/well and then infected with H37Rv or H37Ra for 4 h. BMDM viability was assessed at indicated time points using trypan-blue exclusion assays. The data are presented as the mean ± standard deviation of experiments that were perforemd in triplecate. **(C)** BMDMs were infected with H37Rv at an MOI of 1 for 12 or 72 h. Apoptotic and necrotic cell death were measured using Annexin-V/Propidium iodide (PI) assays by flow cytometry. Bar graphs represent the percentage of *M. tuberculosis*-infected BMDMs that were apoptotic or necrotic cells. **(D)** BMDMs waere infected with H37Rv or H37Ra at MOI of 1 for 48 h and lactate dehydrogenase (LDH) release in the media was determined using a cytotoxicity assay kit. The data are presented as the mean percentage ± standard deviation of experiments that were performed in triplicate. All of the experiments were repeated at least three times with similar results. Significant differences are indicated by ^∗^*P* < 0.05; ^∗∗^*P* < 0.01; ^∗∗∗^*P* < 0.001; and n.s., not significant (*P* > 0.05).

Several studies have shown that the DBA/2 mouse strain is very susceptible to infection with virulent *M. tuberculosis*, whereas the BALB/c and C57BL/6 mouse strains are much more resistant (Medina and North, 1999; Jung et al., 2002; Beamer and Turner, 2005). To investigate differences in *M. tuberculosis* growth between *M. tuberculosis*-infected susceptible and resistant mouse BMDMs, each of the mouse BMDM strains was infected with different infectious doses of virulent H37Rv or attenuated H37Ra, and bacterial growth was measured using CFU assays. After infection with virulent H37Rv, the DBA/2 mouse BMDMs contained approximately twofold more *M. tuberculosis* than was contained in the BALB/c and C57BL/6 mouse BMDMs. However, this was not observed after infection with H37Ra (**Figure 1A**). There was no difference between mouse strains in ingesting bacteria at 4 h after infection when cells were infected with either H37Rv or H37Ra at an MOI of 0.1 or 1 (**Supplementary Figure S1**).

The virulent *M. tuberculosis* strains grew rapidly and induced cell death, such as apoptosis or necrosis, resulting in progressive infection and death in animal models (Park et al., 2006; Porcelli and Jacobs, 2008; Parandhaman and Narayanan, 2014). Each of the mouse BMDM strains was infected with virulent H37Rv or attenuated H37Ra at an MOI of 0.1 or 1, the extracellular bacilli were washed away after infection, and cell growth was assessed at the indicated time points. As shown in **Figure 1B**, the virulent H37Rv strain caused significantly less macrophage growth than the attenuated H37Ra strain in all of the mouse BMDMs. Furthermore, cell growth was significantly more inhibited in the virulent H37Rv-infected DBA/2 BMDMs than in the BALB/c and C57BL/6 BMDMs (**Figure 1B**, left), whereas the numbers of macrophages and related viabilities were similar in cells infected with the attenuated H37Ra strains in all three of the mouse BMDM strains (**Figure 1B**, right). To further dissect the cell death pathways that were initiated by virulent H37Rv infection, each of the BMDM strains that was infected with virulent H37Rv was examined using Annexin V-FITC binding to phosphatidylserine on the cell surface (**Supplementary Figure S2**). At 12 h post-infection, infection with the virulent H37Rv strain consistently resulted in a higher rate of macrophage apoptosis in the DBA/2 BMDMs than the rates observed in the other BMDMs (**Figure 1C**, top). Annexin V^+^, PI^-^ labeling indicates early apoptotic cells, while Annexin V^+^, PI^+^ labeling indicates late apoptotic cells. At 72 h post-infection, the percentage of H37Rv-infected DBA/2 BMDMs that were positive for Annexin-V-FITC and PI binding was still higher than the percentage observed in the other BMDMs (**Figure 1C**, bottom). Furthermore, LDH cytotoxicity was measured higher in H37Rv-infected DBA/2 macrophages at 48 h as shown in **Figure 1D**. The necrotic cell death level was significantly increased in DBA/2 BMDMs when cells were infected with H37Rv at 48 and 72 h. Together, these results suggest that differential susceptibility to virulent *M. tuberculosis* infection is related not only to the control of bacterial growth but also to the induction of host cell death.

### Virulent H37Rv Inhibits ROS Generation in DBA/2 BMDMs

**FIGURE 2:**
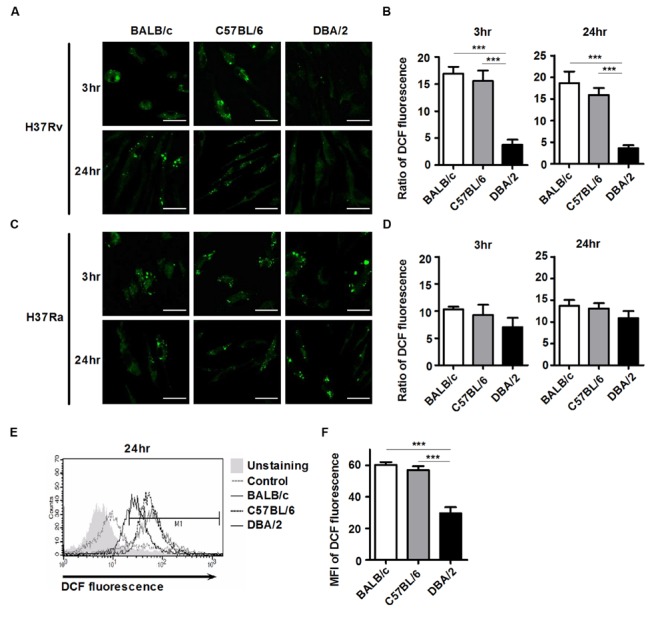
**H37Rv-infected DBA/2 BMDMs generate deficient levels of ROS.** BMDMs were infected with virulent *M. tuberculosis* H37Rv or attenuated *M. tuberculosis* H37Ra (at an MOI of 1) for 3 or 24 h. Cells were labeled with 5-[and-6]-chloromethyl-2′,7′-dichlorodihydro fluorescein diacetate (DCFH-DA) and then measured using confocal microscopy **(A–D)** and flow cytometry **(E,F)**. The bar graph indicates the ratio of DCF fluorescence-positive cells. Ratios were assessed compared to the corresponsding non-infected control cells. The mean ratio ± standard deviation of triplicate measurements are shown (**B,D**; *n* = 6). MFI means mean fluorescence intensity. The mean value ± standard deviation for triplicate measurements are shown **(F)**. All of the experiments were repeated at least three times with similar results. Significant differences are indicated by ^∗∗∗^*P* < 0.001.

Reactive oxygen species generation controls mycobacterial infection and intracellular signaling pathways (Robinson, 2009; Voskuil et al., 2011). To examine whether ROS generation differs between the susceptible and resistant mouse BMDM strains, intracellular ROS levels were monitored using flow cytometry and confocal microscopy as a function of the observed reduction of the redox-sensitive dye, DCFH-DA. As shown in **Figures 2A,B**, infection with virulent H37Rv increased intracellular DCFH-DA fluorescence at 4 h, and this response was maintained at a higher level for up to 24 h in the BALB/c and C57BL/6 BMDMs, but not in the DBA/2 BMDMs. In contrast, during attenuated H37Ra infection, intracellular DCFH-DA fluorescence intensity was increased in all three mouse BMDM strains (**Figures 2C,D**). In line with this result, the flow cytometric analysis also showed that the BALB/c and C57BL/6 BMDMs yielded a stronger DCFH-DA fluorescence signal than the DBA/2 BMDMs after infection with virulent H37Rv (**Figures 2E,F**). These data suggest that the early induction of intracellular ROS determines susceptibility to infection with virulent H37Rv in BMDMs.

### Virulent H37Rv-Infected DBA/2 BMDMs Display Defects in Phagosomal Maturation

**FIGURE 3:**
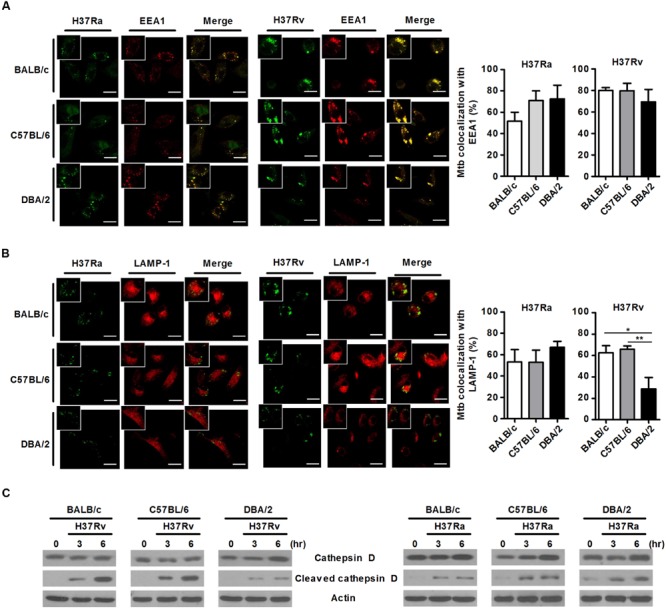
**Virulent H37Rv arrests phagosome maturation in DBA/2 BMDMs.** BMDMs that were isolated from BALB/c, C57BL/6, or DBA/2 mice were infected with virulent H37Rv or attenuated H37Ra for 4 h. After then, cells were fixed and stained with anti-CD107a LAMP-1-Alexa Fluor^®^ 647 or anti-EEA1-Alexa Fluor^®^ 647 antibodies. Co-localization between *M. tuberculosis* and EEA1 **(A)** or LAMP-1 **(B)** was determined using confocal microscopy. The bar graphs shows the percentage of the *M. tuberculosis* foci that co-localized with EEA1 (**A**, top panel) or LAMP-1 (**B**, bottom panel) in the quantitative analysis. The data are expressed as the mean value ± standard deviation of five independent experiments for each condition. Experiments were performed in triplicate. **(C)** Cleaved cathepsin D was detected in H37Rv-infected cells using Western blot assays. Significant differences are indicated by ^∗^*P* < 0.05; ^∗∗^*P* < 0.01.

*M. tuberculosis* can survive and replicate in the phagosomal compartment of infected cells. Following *M. tuberculosis* phagocytosis, several proteins, including Rab5, PI(3)P, early endosomal antigen-1 (EEA-1), Rab7, RILP and LAMP-1, are subsequently recruited to *M. tuberculosis*-containing phagosomes to accelerate the intracellular killing of *M. tuberculosis* (Zhou and Yu, 2008). A number of studies have shown that *M. tuberculosis* can arrest phagosome–lysosome fusion and modulate several events during phagolysosome maturation to avoid destruction by the host’s immune system (Scherr et al., 2009). To assess whether susceptibility to *M. tuberculosis* infection is associated with phagosomal maturation in DBA/2 BMDMs, we measured co-localization between *M. tuberculosis* and EEA1 or LAMP-1 using confocal microscopy in the three mouse BMDM strains. When the BMDMs were analyzed at 3 h after infection, many FITC-labeled *M. tuberculosis* bacteria (both H37Ra and H37Rv) were co-localized with EEA1 in all three mouse BMDM strains (**Figure 3A**). Furthermore, co-localization between LAMP-1- and H37Ra-containing phagosomes was significantly increased in all three of the BMDM types (**Figure 3B**). In marked contrast to these results, the co-localization between the virulent H37Rv strain and LAMP-1 was significantly decreased in DBA/2 BMDMs (**Figure 3B**). However, the level of co-localization was restored in this strain when the cells were pre-treated with IFN-γ (**Figure 5**).

In the final phase of phagosomal maturation, the phagosome acquires V-ATPase and cathepsins (Fratti et al., 2003). Cathepsin D is a soluble lysosomal aspartic endopeptidase that is synthesized in the endoplasmic reticulum as pre-procathepsin D. Procathepsin D is sequentially cleaved to yield a mature and active lysosomal protease under acidic conditions, and this process occurs in late phagosomes (Benes et al., 2008; Vashishta et al., 2009). To further determine whether phagosomal maturation is delayed in virulent H37Rv-infected DBA/2 BMDMs, we measured the levels of cleaved cathepsin D using Western blot analysis in each types of BMDM. The immunoblot results demonstrated that infection with virulent H37Rv led to the processing of procathepsin D to mature cathepsin D (cleaved cathepsin D) in BALB/c and C57BL/6 BMDMs that were infected with virulent H37Rv. However, this process was only weakly induced in H37Rv-infected DBA/2 BMDMs (**Figure 3C**, left). In contrast, when cells were infected with attenuated H37Ra, there was no difference in cleaved cathepsin D expression levels between the three mouse BMDM types (**Figure 3C**, right). These observations indicate that the ability of macrophages to initiate phagocytosis in response to *M. tuberculosis* was not different between the three mouse BMDM types when the cells were exposed to attenuated *M. tuberculosis*. However, the phagosomal maturation process was inhibited in DBA/2 BMDMs, but not BALB/c and C57BL/6 BMDMs, when the cells were exposed to virulent H37Rv.

### Susceptibility to Infection with Virulent *M. tuberculosis* Depends on the Ability to Generate NO

**FIGURE 4:**
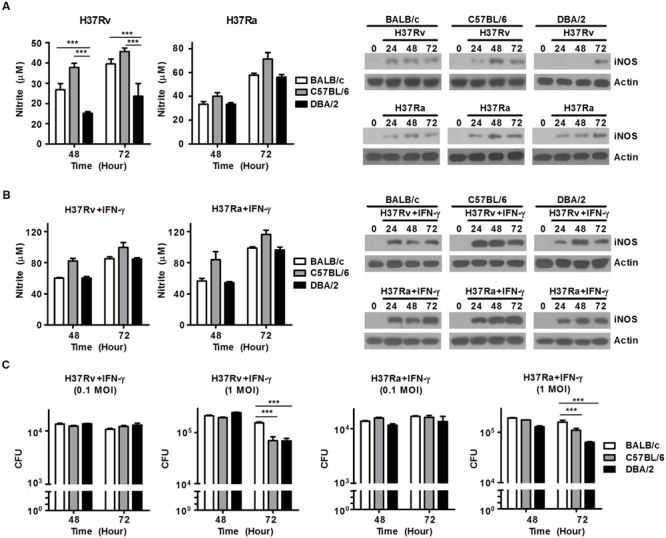
**Treatment with IFN-γ can overcome deficient NO production in DBA/2 BMDMs to control *M. tuberculosis* growth.** BMDMs were infected with virulent H37Rv or attenuated H37Ra for 48 or 72 h. **(A)** The levels of NO that were secreted into the supernatants were measured. The data are presented as the mean ± standard deviation of experiments performed in triplicate (left panel). iNOS levels were measured in the BMDMs using Western blot assays (right panel). **(B,C)** BMDMs were stimulated with IFN-γ (20 ng/ml) after infection with H37Rv or H37Ra. **(B)** The level of NO that was secreted into the supernatants was measured using an NO detection kit. The data are presented as the mean ± standard deviation of experiments that were performed in triplicate (left). iNOS levels were determined in the BMDMs using Western blot assays (right). **(C)** Bacterial growth was confirmed using CFU assessments. The data are presented as the mean ± standard deviation of triplicate experiments. All of the experiments were independently repeated three to five times for each condition with similar results. Significant differences are indicated by ^∗∗∗^*P* < 0.001.

In the murine *M. tuberculosis* infection model, NO is generated in large amounts by macrophages via the activation of inducible nitric oxide synthase (iNOS). The generation of NO controls and restricts the growth of invading mycobacteria (MacMicking et al., 1997; Bosca et al., 2005). To investigate whether the capacity to produce NO is required for resistance to infection with virulent or attenuated *M. tuberculosis* strains in both susceptible (DBA/2) and resistant (C57BL/6 and BALB/c) mouse BMDM strains, we infected BMDMs obtained from BALB/c, C57BL/6, and DBA/2 mice with virulent *M. tuberculosis* H37Rv or attenuated *M. tuberculosis* H37Ra at an MOI of 1 until the indicated time points. We monitored NO production in each BMDM strain. As shown in **Figure 4**, when the BMDMs were infected with virulent *M. tuberculosis* H37Rv, the DBA/2 BMDMs produced significantly lower NO levels than were produced by the BALB/c and C57BL/6 BMDMs. However, this difference was not observed when cells were infected with the attenuated *M. tuberculosis* H37Ra strain. In line with these results, immunoblotting assays further demonstrated that iNOS expression was enhanced in the H37Rv-infected BALB/c and C57BL/6 BMDMs, but not in the H37Rv-infected DBA/2 BMDMs (**Figure 4A**). On the contrary, infection with attenuated H37Ra led to increased iNOS expression levels in all three BMDM strains (**Figure 4A**).

**FIGURE 5:**
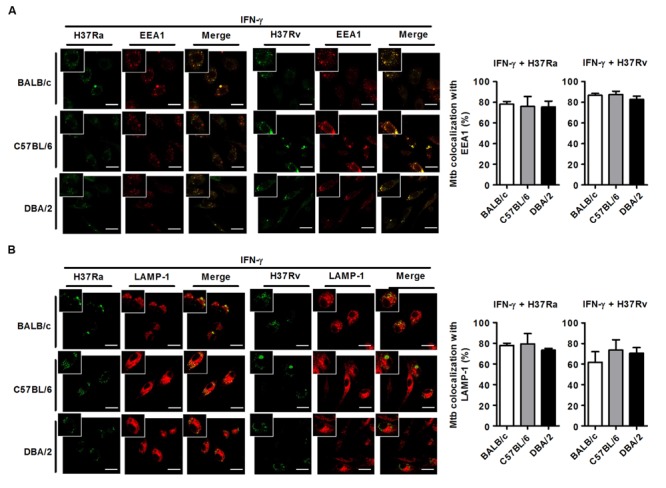
**IFN-γ restores phagosome maturation in H37Rv-infected DBA/2 BMDMs.** BMDMs were stimulated with IFN-γ (20 ng/ml) for 2 h and then infected with either virulent H37Rv or attenuated H37Ra. After 4 h, the cells were fixed and stained with anti-CD107a LAMP-1-Alexa Fluor^®^ 647 or anti-EEA1-Alexa Fluor^®^ 647 antibodies. Co-localization between *M. tuberculosis* and EEA1 **(A)** or LAMP-1 **(B)** was analyzed using confocal microscopy. The bar graphs show the percentage of *M. tuberculosis* foci that co-localized with EEA1 (**A**, top panel) or LAMP-1 (**B**, bottom panel) in the quantitative analysis. The data are presented as the mean ± standard deviation (*n* = 10).

IFN-γ is produced by Th1 cells and natural killer (NK) cells. It is one of the key cytokines that stimulates the activation of macrophages microbicidal activity, which lead to the killing of intracellular bacteria through immune defense mechanisms (Mogues et al., 2001; Jung et al., 2002). In IFN-γ-deficient mice, the growth of *M. tuberculosis* is not controlled, and macrophages are activated at lower rates, suggesting that these mice are extremely susceptible to *M. tuberculosis* infection. Therefore, to confirm whether treatment of IFN-γ can rescue NO secretion in virulent H37Rv-infected DBA/2 BMDMs, BMDMs from all three mouse lines were stimulated with mouse recombinant IFN-γ and monitored over a time course. The DBA/2 BMDMs showed restored NO secretion and iNOS expression to levels similar to those observed in the other BMDM cells following treatment with IFN-γ and infection with either virulent H37Rv or attenuated H37Ra (**Figure 4B**). In addition, treatment of IFN-γ after infection with virulent H37Rv or attenuated H37Ra produced similar growth kinetics between the BALB/c, C57BL/6, and DBA/2 BMDMs (**Figure 4C**). To determine whether the differences that were observed in the ability of the cells to generate NO secretions across the BMDM strains might be related to genetic differences in macrophage function, all three lines of BMDMs were stimulated with IFN-γ for 2 h, and NO production was measured in the cellular supernatants. No difference was observed in the ability of the cells to generate NO following stimulation of IFN-γ (**Supplementary Figure S3**). These data strongly indicate that the failure to produce NO results in susceptibility to infection with virulent *M. tuberculosis* H37Rv, while treatment with IFN-γ restores the ability to produce NO and kill ingested mycobacteria, regardless of the virulence of *M. tuberculosis* strain, in DBA/2 BMDMs.

### The Secretion of TNF-α and IL-10 Is Not Associated with Differential Susceptibility to Infection with Virulent *M. tuberculosis* in All Mouse Strains

**FIGURE 6:**
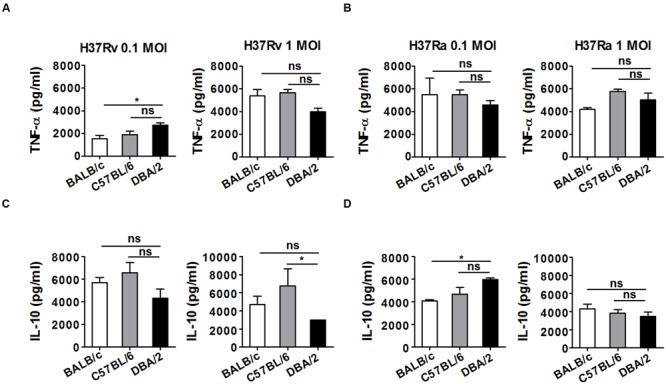
**TNF-α and IL-10 levels were not significantly different in H37Rv-infected DBA/2 BMDMs.** The production of TNF-α **(A,B)** and IL-10 **(C,D)** was measured using ELISA in culture media after BMDMs obtained from three inbred mouse strains were infected with H37Ra or H37Rv. The BMDMs were infected with either attenuated *M. tuberculosis* H37Ra or virulent *M. tuberculosis* H37Rv (at an MOI of 0.1 or 1) for 4 h, and the production of cytokines was anlayzed at 72 h post-infection. Significant differences are indicated by ^∗^*P* < 0.05 and n.s., not significant (*P* > 0.05).

Macrophages are important effector cells that contribute to the production of proinflammatory cytokines, ROS, and NO. These cells therefore contribute to innate immune responses during the early phase of *M. tuberculosis* infection (Raja, 2004). In particular, TNF-α production contributes to the initiation of protective immunity during the early post-infection period (Marino et al., 2010), whereas the increase in IL-10 production that occurs in response to *M. tuberculosis* infection suppresses the protective Th1 response (Redford et al., 2010). To determine whether the observed differences in susceptibility following infection with different *M. tuberculosis* strains might reflect differences in the production of TNF-α or IL-10, we measured the amount of TNF-α or IL-10 that was secreted into the supernatant of each type of BMDM at 24 h after the cells were infected with the virulent *M. tuberculosis* strain H37Rv or the attenuated H37Ra strain at an MOI of 0.1 or 1. As shown in **Figure 6A**, in cells infected with the virulent H37Rv strain at an MOI of 0.1, slightly higher levels of TNF-α were secreted by the DBA/2 BMDMs following infection with H37Rv compared with the BALB/c and C57BL/6 BMDMs, whereas when cells were infected with this strain at an MOI of 1, slightly lower levels of TNF-α were secreted by the DBA/2 BMDMs than the BALB/c and C57BL/6 BMDMs. There was no difference in the amount of TNF-α that was secreted by the three BMDM strains following infection with the attenuated H37Ra strain (**Figure 6B**). Therefore, while the amount of secreted TNF-α appears to be dependent on the infectious dose when cells are infected with virulent H37Rv, this does not appear to be the case when cells are infected with attenuated H37Ra infection. In addition, when cells were infected with the H37Rv strain at MOI of 1, lower IL-10 levels were detected in the DBA/2 BMDM supernatants than in the C57BL/6 BMDM supernatants (**Figure 6C**). In contrast, among the mouse BMDM types, IL-10 levels were slightly higher in the DBA/2 BMDMs following infection with the attenuated H37Ra strain at an MOI of 0.1 (**Figure 6D**). These results suggest that the differential induction of either pro-inflammatory or anti-inflammatory cytokines is not correlated with differential susceptibility to virulent and attenuated *M. tuberculosis* strains in *M. tuberculosis*-resistant and -susceptible mouse macrophages.

## Discussion

Tuberculosis is caused by *M. tuberculosis*, which infects approximately one-third of the world’s population. However, many infected individuals remain asymptomatic with only 5–10% of infected people developing active tuberculosis (Young et al., 2008). The factors that determine susceptibility to *M. tuberculosis* infection remain unknown (Lin and Flynn, 2010). Mice are one of the most popular and widely used experimental animal models of *M. tuberculosis* infection (Chackerian and Behar, 2003). Susceptibility to *M. tuberculosis* infection in mice is dependent upon the genetic background of inbred mouse strains, although all inbred mouse strains can be easily infected with *M. tuberculosis*, which induces a Th1 response and lung pathology (Mitsos et al., 2000).

We previously demonstrated that the C57BL/6 and BALB/c strains have longer survival times than DBA/2 strains following infection with the virulent *M. tuberculosis* strain H37Rv (Jung et al., 2009), indicating that C57BL/6 and BALB/c mice are more resistant to infection with virulent *M. tuberculosis* than DBA/2 mice. However, despite the superior resistance that BALB/c mice possess compared to DBA/2 mice, BALB/c and DBA/2 mice generate a similar number of Th1 cells. Furthermore, iNOS expression was increased in *M. tuberculosis*-infected lung sections obtained from each mouse, suggesting that susceptibility to virulent *M. tuberculosis* infection is not governed by the ability to generate a Th1 response. In this report, we sought to correlate the ability to generate an innate immune response with genetic susceptibility during the very early phase of infection, when the number of *M. tuberculosis* bacilli in macrophages is limited. Here, we demonstrate that after infection with the virulent *M. tuberculosis* H37Rv strain, macrophages that were isolated from C57BL/6 and BALB/c mice inhibited *M. tuberculosis* growth better than macrophages isolated from DBA/2 mice. However, the growth of the attenuated *M. tuberculosis* H37Ra strain was similar across the three mouse macrophage strains.

*M. tuberculosis*-infected macrophages undergo various types of cell death, including apoptosis, necrosis, and autophagy. These forms of cell death are associated with dramatically different outcomes during an infection. Apoptotic cell death in *M. tuberculosis*-infected macrophages is related to mycobacterial killing, whereas necrotic cell death is associated with infection transmission (Danelishvili et al., 2003; Abebe et al., 2011). Recent studies have shown that *M. tuberculosis* virulence determines the type of host cell death and regulates the balance between apoptosis and necrosis (Butler et al., 2012). In contrast, other studies have demonstrated that cell death in *M. tuberculosis*-infected macrophages is not dependent upon bacterial virulence (Beisiegel et al., 2009). In the present study, infection with virulent H37Rv led to cell death, especially via apoptosis and necrosis, and a massive cell death ratio was observed in the H37Rv-infected DBA/2 BMDMs. Although apoptosis seemed to be the dominant cell death pathway in DBA/2 macrophages at 12 h after infection, necrotic cell death was significantly increased at 72 h in the same macrophages when the cells were infected with H37Rv. Therefore, DBA/2 macrophages display a similar level of defense against attenuated *M. tuberculosis* strains, but they display defects in their bacteriostatic and survival mechanisms when exposed to a more virulent strain of *M. tuberculosis*.

Macrophages generate ROS from O_2_ using NADPH oxidase, NOX2, to kill invading microorganisms (Chan et al., 2001). NOX2 activity then induces ROS production in phagosomes. *In vivo* experiments demonstrated that mice deficient in Phox partially inhibited the growth of *M. tuberculosis* in an aerosolized infection model, suggesting a role for ROS in controlling *M. tuberculosis* during the early phase of infection (Jung et al., 2002). Recent studies have shown that the *M. tuberculosis* growth was greatly increased in gp91phox^-/-^ mice or siNOX2-transfected cells (Yang et al., 2009), suggesting that ROS influence the control of *M. tuberculosis* growth. In agreement in this finding, the present study demonstrated that infection with virulent H37Rv induced ROS generation in mouse macrophages. However, lower ROS production levels were observed in H37Rv-infected DBA/2 BMDMs during the early phases of infection, indicating that these cells did not induce the full ROS response as the defense mechanisms against virulent *M. tuberculosis*.

Pathogens have evolved different mechanisms to allow survival in macrophages. For instance, *Listeria* and *Shigella* sp. escape into the cytoplasm to avoid degradation (Corr and O’Neill, 2009; Dupont et al., 2009), whereas *Legionella* and *Mycobacterium* sp. disrupt the phagosomal maturation process (Corr and O’Neill, 2009). Following the internalization of *M. tuberculosis* into macrophages, *M. tuberculosis* inhibits the acidification of phagosomes by interfering with the fusion of phagosomes and lysosomes and modulating key events in host signaling pathways (Scherr et al., 2009). In this study, we confirmed that there was no difference in the number of foci in which we observed co-localization between EEA1 and virulent H37Rv-containing vacuoles across the three mouse BMDM strains. In contrast, the number of foci that represented co-localization between LAMP-1 and virulent H37Rv-containing vacuoles was significantly lower in DBA/2 BMDMs. However, these results were not observed when we infected BMDMs with the attenuated H37Ra strain. A recent study showed that while virulent *M. tuberculosis* strains interrupted the activity of Rab GTPases and LAMPs (Sun et al., 2013), both of which regulate phagosomal maturation, attenuated *M. tuberculosis* strains did not have this effect. In accordance with the results of this study, although all of the BMDM strains showed similar phagocytic abilities in the early infection phase, the DBA/2 BMDMs failed to generate mature phagosomes, and there were fewer acidic vacuoles in DBA/2 BMDMs than in C57BL/6 or BALB/c BMDMs. Phagosomes containing microbes exchange membrane components between phagosomes and endosomes, which allows interactions between various components of the endosomal pathway. These events result in the acidification of phagosomes via the activation of vacuolar type-H^+^ ATPase pumps (V-H^+^ ATPase) and lysosomal hydrolases, such as cathepsin D (Luzio et al., 2007). Cathepsin D is cleaved into its mature form under acidic conditions, and this process is initiated only in late stage phagosomes (Turk et al., 2012). In this study, much lower levels of mature cathepsin D were expressed in H37Rv-infected DBA/2 BMDMs than in BALB/c and C57BL/6 BMDMs.

Recent studies have shown that the levels of iNOS expression the lung are similar between the three mouse strains at day 30 post-infection (Jung et al., 2009). However, in our study, much lower levels of NO were produced in the DBA/2 macrophages than in the C57BL/6 or BALB/c macrophages when the cells were infected with the H37Rv strain, suggesting that macrophages from DBA/2 mice are more susceptible to virulent *M. tuberculosis* infection during the innate phase of infection. CD4 T cells, CD8 T cells and NK cells secrete IFN-γ during *M. tuberculosis* infection, and IFN-γ subsequently induces the expression of the antimicrobial enzyme iNOS, which activates the bactericidal functions of macrophages (Pearl et al., 2001; Kahnert et al., 2006). Several reports have shown that IFNγ- or iNOS-deficient mice that were infected with *M. tuberculosis* die quickly and exhibit uncontrolled *M. tuberculosis* growth, indicating that IFN-γ is an essential component to activate the immune responses that limit *M. tuberculosis* growth following infection (Cooper et al., 1993; Jung et al., 2002). Although, there was no difference in the ability of BMDMs to generate NO between the three mouse lines examined in this study, when the macrophages were activated with IFN-γ, DBA/2 macrophages displayed defective capacity to produce NO during the very early period following infection. However, these macrophages were able to generate NO when they were activated with IFN-γ during the sustained phase of adaptive immunity. Moreover, there was no difference in the induction of NO production in BMDMs that were infected with attenuated H37Ra. These results indicate that virulent *M. tuberculosis* controls host defense response to increase its ability to survive intracellularly and that is uses a variety of mechanisms to evade host responses.

TNF-α is a pro-inflammatory cytokine with anti-mycobacterial effects (Flynn et al., 1995). Recently, treatment with recombinant TNF-α in the presence of IFN-γ was found to enhance mycobacterial killing (Herbst et al., 2011), whereas defects in TNF-α or its receptor were found to promote *M. tuberculosis* replication and progressive lung disease in *M. tuberculosis*-infected mice (Mohan et al., 2001). These data suggest that TNF-α plays a critical role in the innate immune response to *M. tuberculosis* infection. In contrast, IL-10 is considered to be an anti-inflammatory cytokine that suppress macrophages during *M. tuberculosis* infection, which specifically downregulate IL-12 production and inhibits antigen presentation activity (Redford et al., 2010, 2011). IL-10, therefore, counteracts the macrophage-activating ability of IFN-γ. Our study reveals that higher levels of TNF-α were observed in macrophages that were exposed to virulent H37Rv at an MOI of 1 than in cells infected with the same strain at an MOI of 0.1. Another study showed that IFNγ-activated BMDMs became apoptotic in an NO-dependent manner (Albina et al., 1993). In contrast, others have shown that the induction of apoptosis in macrophages is independent of NO, and it mediated by TNF-α, which induces mycobacterial killing activity (Herbst et al., 2011). In line with this result, we found that infection with virulent *M. tuberculosis* induced an increase in cell death in DBA/2 BMDMs in an NO-dependent manner.

## Conclusion

Our study is the first to show that DBA/2 BMDMs are unable to control the growth of virulent *M. tuberculosis* even when the infectious dose is very low (e.g., an MOI of 0.1 or 1). This inability resulted from the insufficient generation of innate immune responses, including a deficit in the amount of ROS and NO that was generated, and an inadequate level of phagosomal maturation. In addition, our study suggests that defects in the initiation of innate immune responses may determine susceptibility to infection with virulent H37Rv, but they are not associated with susceptibility to attenuated H37Ra during the very early phase of *M. tuberculosis* infection.

## Author Contributions

Y-JJ designed the experiments, analyzed the data and wrote the manuscript with contributions from all of the co-authors. H-JL performed most of the experiments and statistical analysis. H-JK analyzed the data and provided reagents and advice during the FACS analysis.

## Conflict of Interest Statement

The authors declare that the research was conducted in the absence of any commercial or financial relationships that could be construed as a potential conflict of interest.

## Supplementary Material

FIGURE S1 **The ability of ingesting bacteria is not different between three strains of BMDM during *M. tuberculosis* infection for 4 h.** 2 × 10^5^ BMDMs were infected with H37Rv or H37Ra at MOI of 0.1 or 1 for 4 h. And then, the viability of intracellular bacteria was assayed based on the number of CFUs that were observed after 3 weeks. The data are presented as the mean ± standard deviation of triplicate experiments. Significant differences are indicated by n.s., not significant (*P* > 0.05).

FIGURE S2 **Cell death populations in uninfected BMDMs and H37Rv-infected BMDMs at MOI of 0.1.** Each BMDMs were incubated with antibiotic-free medium **(A)** or infected with H37Rv at an MOI of 0.1 **(B)** for 12 or 72 h. The portion of death population was measured using an Annexin-V/Propidium iodide (PI) assay by flow cytometry.

FIGURE S3 **There was no difference between the three mouse strains in the ability of IFNγ-stimulated BMDMs to generate NO.** BMDMs isolated from three inbred mouse strains were stimulated with IFN-γ (20 ng/ml) for 2 h, and the level of NO secreted into the culture supernatnat was determined in each cells. Significant differences are indicated by n.s., not significant (*P* > 0.05).
