# Supplementary figures and images for: Insufficient Generation of Mycobactericidal Mediators and Inadequate Level of Phagosomal Maturation Are Related with Susceptibility to Virulent Mycobacterium tuberculosis Infection in Mouse Macrophages
Source: Front Microbiol. 2016 Apr 18;7:541. doi: 10.3389/fmicb.2016.00541 (PMC4834433; doi:10.3389/fmicb.2016.00541)

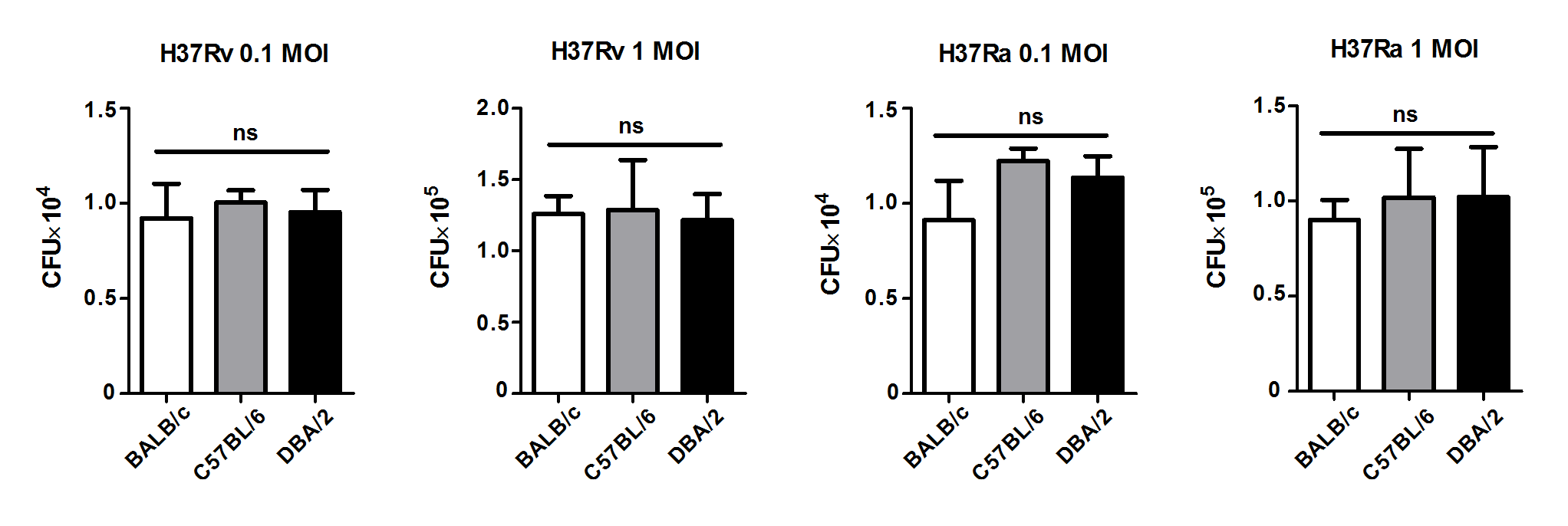

Supplement: FIGURE S1 — The ability of ingesting bacteria is not different between three strains of BMDM during M. tuberculosis infection for 4 h. 2 × 10^5^ BMDMs were infected with H37Rv or H37Ra at MOI of 0.1 or 1 for 4 h. And then, the viability of intracellular bacteria was assayed based on the number of CFUs that were observed after 3 weeks. The data are presented as the mean ± standard deviation of triplicate experiments. Significant differences are indicated by n.s., not significant (P > 0.05). [file Image_1.TIF]

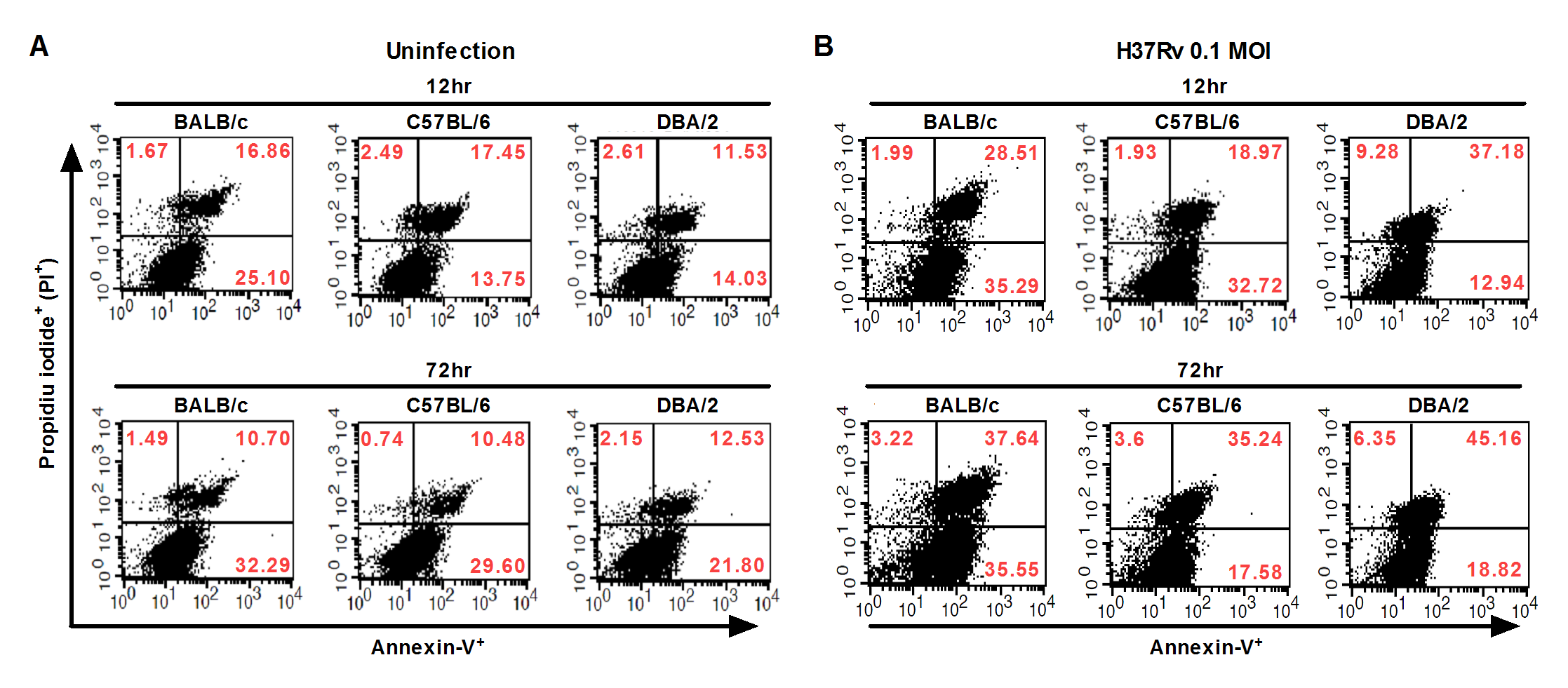

Supplement: FIGURE S2 — Cell death populations in uninfected BMDMs and H37Rv-infected BMDMs at MOI of 0.1. Each BMDMs were incubated with antibiotic-free medium (A) or infected with H37Rv at an MOI of 0.1 (B) for 12 or 72 h. The portion of death population was measured using an Annexin-V/Propidium iodide (PI) assay by flow cytometry. [file Image_2.TIF]

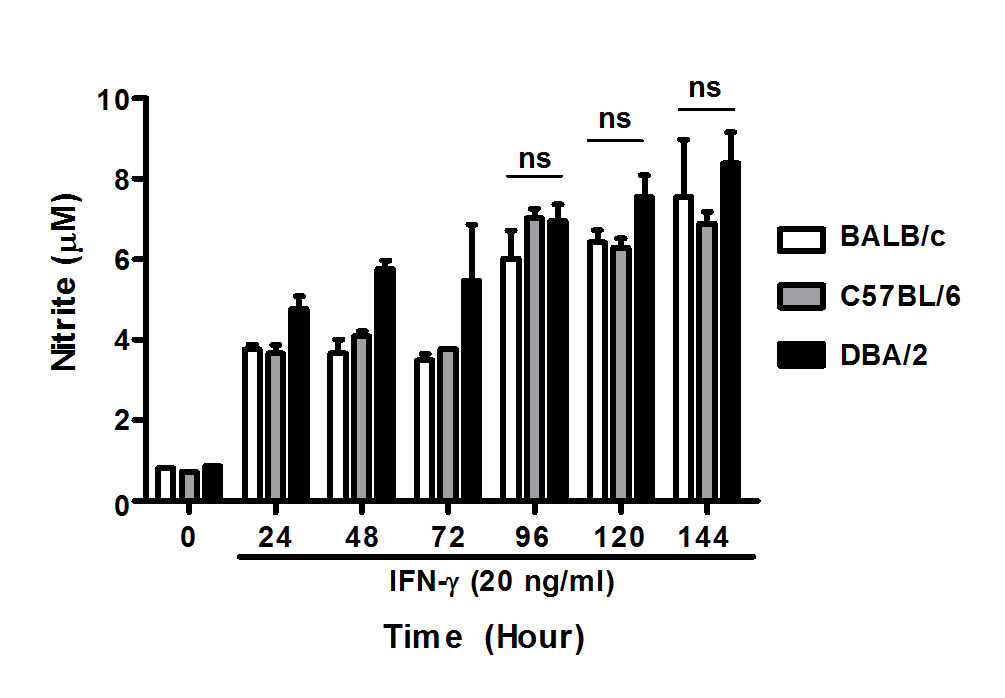

Supplement: FIGURE S3 — There was no difference between the three mouse strains in the ability of IFNγ-stimulated BMDMs to generate NO. BMDMs isolated from three inbred mouse strains were stimulated with IFN-γ (20 ng/ml) for 2 h, and the level of NO secreted into the culture supernatnat was determined in each cells. Significant differences are indicated by n.s., not significant (P > 0.05). [file Image_3.TIF]
